# Genomic selection for lentil breeding: Empirical evidence

**DOI:** 10.1002/tpg2.20002

**Published:** 2020-03-27

**Authors:** Teketel A. Haile, Taryn Heidecker, Derek Wright, Sandesh Neupane, Larissa Ramsay, Albert Vandenberg, Kirstin E. Bett

**Affiliations:** ^1^ Dep. of Plant Sciences Univ. of Saskatchewan 51 Campus Drive Saskatoon SK S7N 5A8 Canada

## Abstract

Genomic selection (GS) is a marker‐based selection initially suggested for livestock breeding and is being encouraged for crop breeding. Several statistical models are used to implement GS; however, none have been tested for use in lentil (*Lens culinaris* Medik.) breeding. This study was conducted to compare the accuracy of different GS models and prediction scenarios based on empirical data and to make recommendations for designing genomic selection strategies for lentil breeding. We evaluated nine single‐trait (ST) models, two multiple‐trait (MT) models, and a model that incorporates genotype × environment interaction (GEI) using populations from a lentil diversity panel and two recombinant inbred lines (RILs). The lines in all populations were phenotyped for five phenological traits and genotyped using a custom exome capture assay. Within‐population, across‐population, and across‐environment genomic predictions were made. Prediction accuracy varied among the evaluated models, populations, prediction scenarios, and traits. Single‐trait models showed similar accuracy in the absence of large effect quantitative trait loci (QTL) but BayesB outperformed all models when there were QTL with relatively large effects. Models that accounted for GEI and MT‐GS models increased prediction accuracy for a low heritability trait by up to 66 and 14%, respectively. Moderate to high accuracies were obtained for within‐population (range of .36–.85) and across‐environment (range of .19–.89) predictions but across‐population prediction accuracy was very low. Results suggest that GS can be implemented in lentil breeding to make predictions within populations and across environments, but across‐population prediction should not be considered when the population size is small.

**Abbreviations**
DTF: days to flowering
DTM: days to maturity
DTS: days to swollen pods
G‐BLUP: genomic best linear unbiased prediction
GEBV: genomic estimated breeding value
GEI: genotype × environment interaction
GS: genomic selection
LD: linkage disequilibrium
LDP: lentil diversity panel
MT: multiple‐trait; REP, reproductive period
RIL: recombinant inbred line
RKHS: Bayesian reproducing kernel Hilbert spaces regression
RR‐BLUP: ridge regression best linear unbiased prediction
QTL: quantitative trait loci
ST: single‐trait
TP: training population
VEG: vegetative period

## 1 INTRODUCTION

Lentil is a diploid (2*n* = 14), self‐pollinating crop with a haploid genome size of approximately 4 Gb (Arumuganathan & Earle, 1991). It is an important pulse crop, which provides a dietary source of protein, carbohydrate, micronutrients, vitamins, and fiber. Lentil is used increasingly as an important source of protein for people whose diet is solely plant based. It has ecological advantages as a rotational crop in cereal‐based cropping systems by enabling better management of pests, herbicide residue, and soil nitrogen. In 2017, lentil was grown on more than 6.6 Mha across 52 countries, accounting for an annual production of 7.6 Mt (FAO, 2019). Until recently, lentil has received little attention in terms of genetic research. Advances in high‐throughput genotyping technologies and a relative decline in sequencing costs have resulted in the availability of abundant single nucleotide polymorphisms (SNPs) covering the gene space of lentil through in‐depth sequencing of the entire set of exons in the genome (Ogutcen, Ramsay, von Wettberg, & Bett, 2018). This development opened new opportunities to evaluate genomic tools such as GS for lentil improvement.

Genomic selection is a marker‐based selection that uses a training population (TP) that is phenotyped and genotyped to train a statistical model. The model is then used to predict genomic estimated breeding values (GEBVs) of individuals in a breeding population that has been genotyped but not phenotyped (Meuwissen, Hayes, & Goddard, 2001). The GEBVs are then used to select superior lines rather than the actual phenotypes. Model prediction accuracy is tested using independent lines in a validation population for which phenotypic and genotypic data are available. This is commonly achieved by partitioning the same population into training and validation sets in which GEBVs are predicted for individuals in the validation set. The correlation between predicted values and the observed phenotypes is considered the prediction accuracy.

Unlike the standard marker‐assisted selection, which uses a small number of markers associated with major QTL, GS uses a large set of genome‐spanning markers to potentially capture all the QTL underlying a trait (Meuwissen et al., 2001). Fitting all markers simultaneously avoids multiple testing and the need to identify marker–trait associations based on an arbitrarily chosen significance threshold. Genomic selection was initially suggested for livestock breeding (Meuwissen et al., 2001) and was later evaluated and implemented in crop breeding, especially for wheat (*Triticum* sp.) and maize (*Zea mays* L.) (Bernardo, 2009; Bernardo & Yu, 2007; Beyene et al., 2015; Crossa et al., 2016; Crossa et al., 2014). The goal of GS is to enhance the genetic gain of quantitative traits by accelerating the breeding cycle and increasing selection intensity (Battenfield et al., 2016; Heffner, Lorenz, Jannink, & Sorrells, 2010). Thus far, there is no information on the utility of GS for lentil breeding.

The accuracy of GS is affected by several factors, including TP size, marker density, trait heritability, the genetic relationship between the training and breeding populations, linkage disequilibrium (LD), population structure, GEI, and the statistical model used (Burgueño, de los Campos, Weigel, & Crossa, 2012; Calus, 2010; Combs & Bernardo, 2013; de los Campos, Veturi, Vazquez, Lehermeier, & Pérez‐Rodríguez, 2015; Heffner, Jannink, & Sorrells, 2011; Jarquín et al., 2014; VanRaden et al., 2009; Wientjes, Veerkamp, & Calus, 2013). A large TP size, higher heritability, and higher marker density usually improve genomic prediction accuracy (Asoro, Newell, Beavis, Scott, & Jannink, 2011; Heffner et al., 2011; Liu et al., 2018; Meuwissen et al., 2001). However, uniform marker distribution across the genome is essential to tag important QTL and maintain GS accuracy (Bassi, Bentley, Charmet, Ortiz, & Crossa, 2016). The main assumption of GS is that each of the QTL is in LD with at least one nearby marker, and potentially all the genetic variance can be explained by the markers (Calus, 2010; Goddard & Hayes, 2007). The effect of population structure on prediction accuracy varies based on prediction strategies and the genetic architectures of traits and populations (Guo et al., 2014). It has also been shown that incorporating GEI into GS models can improve the accuracy of prediction (Crossa et al., 2015; de los Campos et al., 2015; Jarquín et al., 2017; Lopez‐Cruz et al., 2015). Therefore, it is important to evaluate models that account for GEI in each target environment.

**Core Ideas**

- BayesB showed the highest prediction accuracy for traits controlled by few QTL with relatively large effects.
- Incorporating genotype × environment interactions improved prediction accuracy by up to 66%.
- Genomic selection can be implemented in lentil to make within‐population and across‐environment predictions.
- Across‐population genomic prediction should not be considered in lentil when population size is small.

Genomic selection models fit a large number of markers on a small number of phenotypic observations, which may lead to overfitting when markers are treated as fixed effects (de los Campos, Hickey, Pong‐Wong, Daetwyler, & Calus, 2013). Standard GS models treat markers as a random effect and reduce the dimension of the marker data by either selecting markers, shrinking marker effect estimates, or a combination of both (de los Campos et al., 2013). Several methods, including ridge regression, Bayesian regression, kernel‐based approaches, and machine learning algorithms, have been developed that differ mainly in their assumptions about the variances of marker effects (Breiman, 2001; Gianola & van Kaam, 2008; Gianola, Fernando, & Stella, 2006; Habier, Fernando, Kizilkaya, & Garrick, 2011; Meuwissen et al., 2001; Park & Casella, 2008; Pérez, de los Campos, Crossa, & Gianola, 2010). Ridge regression assumes that all loci explain equal amounts of the genetic variance, while Bayesian models allow the variance to vary across loci (Meuwissen et al., 2001). The assumption of common variance for all loci in a ridge regression is unrealistic and may underestimate the effects of known major QTL. Alternatively, models have been developed that treat markers associated with traits, markers tagging candidate genes, and previously discovered QTL as fixed effects (Spindel et al., 2016). Initially, GS models were developed for predicting a single trait from a single environment. In recent years, multivariate models and models that account for GEI have been developed (Burgueño et al., 2012; Jarquín et al., 2014; Jia & Jannink, 2012; Jiang et al., 2015; Lopez‐Cruz et al., 2015). Multivariate and multi‐environment models use the correlation of traits and environments to enhance GS accuracy (Jia & Jannink, 2012; Lopez‐Cruz et al., 2015). To date, none of these models have been tested in lentil mainly due to the lack of dense genetic resources. The objectives of this study were to compare the accuracy of different GS models and prediction scenarios based on empirical data in lentil and to make recommendations for designing genomic selection strategies for lentil breeding.

## 2 MATERIALS AND METHODS

### 2.1 Plant material and phenotyping

We used three populations to evaluate genomic prediction accuracy in lentil (Supplemental Table S1). The first population was a lentil diversity panel (LDP) composed of 324 accessions obtained from the gene banks of the International Center for Agricultural Research in the Dry Areas (ICARDA), the USDA, Plant Gene Resources of Canada (PGRC), and cultivars developed at the Crop Development Centre (CDC), University of Saskatchewan (https://knowpulse.usask.ca/Lentil-Diversity-Panel). This population was evaluated at Sutherland (52°9′ N, 106°30′ W) from 2016 to 2018 and Rosthern, SK (52°41′ N, 106°17′ W) in 2016 and 2017. The second population, LR‐01, was composed of 110 RILs developed from a cross between ILL 1704 and ‘CDC Robin’. CDC Robin is a red cotyledon lentil cultivar developed at the CDC, and ILL 1704 is a landrace from Ethiopia (Vandenberg et al., 2002). The third population, LR‐11, included 120 RILs developed from a cross between ‘ILL 8006‐BM4’ and ‘CDC Milestone’. CDC Milestone is a yellow cotyledon lentil cultivar developed at the CDC (Vandenberg et al., 2001), and ‘ILL 8006‐BM4’ was derived from ‘Barimasur‐4’, a red cotyledon cultivar from Bangladesh (Sarker, Erskine, Hassan, Afzal, & Murshed, 1999). The LR‐11 population was evaluated at Sutherland and Rosthern, SK, in 2017 and 2018. LR‐01 was grown at the same two locations in 2018. For all populations, field trials were established in 1‐m^2^ three‐row microplots arranged in a randomized complete block design with three replications in each site‐year. Seeding rates of 60, 100, and 120 seeds m^−2^ were used for the LDP, LR‐01, and LR‐11, respectively. Plots were seeded between late April and mid‐May and harvested between mid‐August and early September in each year.

The phenotypic traits measured in all populations included days to flowering (DTF), vegetative period (VEG), days to swollen pods (DTS), days to maturity (DTM), and reproductive period (REP). Days to flowering and DTS were recorded when 10% of the plants in a plot had at least one open flower and pods with fully swollen seeds, respectively. The trait DTM was recorded as the date when 10% of the plants displayed 50% pod maturity. Vegetative and reproductive periods were recorded as the number of days from emergence to flowering and from flowering to maturity, respectively. The phenotypic data were analyzed using analysis of variance (ANOVA) with SAS mixed models, version 9.4 (SAS Institute, 2015). For each population, the phenotypic data were analyzed separately for each environment (site‐year) and combined across environments. Genotype was considered a fixed effect and replication was considered random for the data analysis of each environment. For combined data analyses, replication nested in environment, environment (site‐years), and GEI were considered random effects. The Kenward–Roger degrees of freedom approximation method was used to compute the degrees of freedom for means (Kenward & Roger, 1997). Broad‐sense heritability (*H* ^2^) on a plot basis was estimated for all traits in each population using the equation σ_g_^2^/(σ_g_^2^ + σ_ge_^2^/*e* + σ_ε_^2^/*re*), where σ_g_^2^ is the genetic variance, σ_ge_^2^ is the GEI variance, σ_ε_^2^ is the residual variance, *e* is the number of environments, and *r* is the number of replications per environment. Variance components were estimated in SAS using the restricted maximum likelihood method described by Holland, Nyquist, and Cervantes‐Martínez (2003), with genotype, environment, GEI, and replication considered as random effects.

### 2.2 DNA extraction and genotyping

Genomic DNA was extracted from fresh leaves of 2‐ to 3‐wk‐old seedlings for all lines using a Qiagen MiniPrep Kit (Qiagen). Genotyping of all lines was performed using a custom exome capture assay as described by Ogutcen et al. (2018). Markers with more than 5% missing data and a minor allele frequency of less than 5% were removed prior to analysis (Tabangin, Woo, & Martin, 2009). A total of 9394, 24,395, and 39,297 markers that were common between the LDP and LR‐11, the LDP and LR‐01, and LR‐01 and LR‐11, respectively, were used for genomic prediction. Missing marker genotypes were imputed by replacing them with the population mean for that marker using the function A.mat in the R package rrBLUP, version 4.4 (Endelman, 2011).

### 2.3 Statistical models and prediction scenarios

#### 2.3.1 Standard single‐trait prediction models

Single‐trait predictions were made using ridge regression best linear unbiased prediction (RR‐BLUP), genomic best linear unbiased prediction (G‐BLUP), BayesA, BayesB, BayesCπ, Bayesian Lasso, Bayesian ridge regression, and Bayesian reproducing kernel Hilbert spaces regression (RKHS). The RKHS regression is a semi‐parametric approach that accounts for both additive and nonadditive genetic effects (de los Campos, Gianola, & Rosa, 2009), while all the other models are based only on the additive effect. The basic equation for the single trait models is

(1) y=Xβ+Zu+ε

where **y** is a vector of observed phenotypes, **X** is a design matrix for fixed effects, **β** is a vector of fixed effects, **Z** is a design matrix for random effects, **u** is a vector of random effects, and **ε** is a vector of residuals.

All models were fitted in R (R Core Team, 2016), using the Bayesian generalized linear regression (BGLR) package, version 1.0.8 (Pérez & de los Campos, 2014), and the rrBLUP package, version 4.4 (Endelman, 2011). The default settings of BGLR (five degrees of freedom and the scale parameter based on the sample variance of the phenotypes) were used (Pérez & de los Campos, 2014). For G‐BLUP, the genomic relationship matrix was computed according to VanRaden (2008). For RKHS, we used a Gaussian kernel with a bandwidth parameter of *h* = 1/*M* × {1/5, 1, 5} as described by Pérez and de los Campos (2014), where *M* is the median squared Euclidean distance between all lines calculated using off‐diagonals.

#### 2.3.2 Genomic selection model that combines genome‐wide association studies

We used a GS+GWAS model to fit significant markers identified from fold‐specific genome‐wide association studies (GWAS) as fixed effects in GS (Spindel et al., 2016). GS+GWAS is equivalent to RR‐BLUP when no marker is fitted as a fixed effect (Spindel et al., 2016). GS+GWAS is similar to the model in Equation (1) except that the allele dosages of the markers fitted as fixed effects were added as columns to the **X** matrix and then excluded from calculation of the genomic relationship matrix (Spindel et al., 2016). The accuracy of GS+GWAS was tested using a five‐fold cross‐validation design. In the LDP, GWAS was conducted in each fold based on the phenotypic and genotypic data of the TP using the compressed mixed linear model in GAPIT, version 3.0 (Lipka et al., 2012). Population structure and cryptic relatedness were accounted for using five marker‐derived principal components and kinship as covariates. For LR‐01 and LR‐11, single marker regression was performed in each fold based on the phenotypic and genotypic data of the TP using the *lm* function in R. For all three populations, the *P* values were sorted from low to high, and multiple testing correction was performed based on a false discovery rate using the function p.adjust and the BH method in R (Benjamini & Hochberg, 1995). Then the SNPs on each chromosome were binned into 500‐kb distance, and the SNP with the lowest *P* value was extracted from each bin. This step was performed to avoid fitting adjacent SNPs tagging the same QTL as fixed effects. Up to three most significant markers (false discovery rate of 0.1) were selected separately for each fold in the five‐fold cross‐validation method. When no marker met this threshold, only the most significant marker was selected. The selected markers were then included in the GS+GWAS model as fixed effects, while all the remaining markers from the full marker density were included as random effects. The GS+GWAS model was fitted in R using the kinship.BLUP function in the rrBLUP package (Endelman, 2011).

#### 2.3.3 A reaction norm model that accounts for genotype × environment interaction

The effect of modeling GEI on genomic prediction accuracy was assessed using a reaction norm model that incorporates the main effects and interactions of molecular markers and environments using covariance structures (EG‐G × E) as described by Jarquín et al. (2014). The main effects of markers and environments were included in the model using techniques similar to the standard G‐BLUP, while the interaction terms were modeled using a cell by cell product of two covariance structures, [**Z_g_GZ**′**_g_**] ◦ [**Z_E_Z**′**_E_**], where **Z_g_** and **Z_E_** are the incidence matrices for lines and environments, respectively; **G** is a marker‐derived genomic relationship matrix; and ◦ indicates the cell by cell (Hadamard) product between two matrices (Jarquín et al., 2014). In this model, phenotypes (*y_ijk_*) are described as the sum of an overall mean (μ) plus a random deviation due to the environment (*E_i_*), which is a combination of site‐years plus marker covariates using a marker‐derived genomic relationship matrix (**g*_j_***), plus an interaction term between genotypes and environments (g*E_ij_*) and a residual term (ε*_ijk_*):

(2) $$y_{ijk}\;=\;\mu \;+\;E_{i}\;+\;g_{j}\;+\;gE_{ij}\;+\;\varepsilon_{ijk}$$

with *E_i_* ∼ *N*(0,σ_E_^2^), g ∼ *N*(0,**G**σ_g_^2^), g*E* ∼ *N*(0,[**Z_g_GZ**′**_g_**] ◦ [**Z_E_Z**′**_E_**]σ_gE_^2^), and ε*_ijk_* ∼ *N*(0,σ_g_^2^).

The accuracy of the EG‐G × E model was compared with a standard G‐BLUP model plus a random environmental effect (*E*), yielding the EG model without the interaction term:

(3) $$y_{ijk}\;=\;\mu \;+\;E_{i}\;+\;g_{j}\;+\;\varepsilon_{ijk}$$

with *E_i_* ∼ *N*(0,σ_E_^2^), g ∼ *N*(0,**G**σ_g_^2^), and ε*_ijk_* ∼ *N*(0,σ_g_^2^).

Prediction accuracy of the reaction norm model was assessed using two cross‐validation designs (CV1 and CV2) as implemented by Burgueño et al. (2012). The first cross‐validation design (CV1) involved predicting phenotypes of lines that have never been tested in any of the environments (newly developed lines). The second design (CV2) involved predicting phenotypes of lines that were evaluated in some environments but not in others (i.e., incomplete field trials). CV1 assigns lines to folds, and therefore records of the same line are assigned to the same fold; in CV2, individual phenotypes of each line were assigned to folds, and thereby phenotypes of a line from different environments are potentially assigned to different folds (Jarquín et al., 2014; Pérez‐Rodríguez et al., 2015). Both CV1 and CV2 were implemented in a five‐fold design.

### 2.4 Multiple‐trait prediction models

We used MT‐BayesA and MT‐BayesA matrix models for joint prediction of multiple traits (Jiang et al., 2015). The equation for the multiple‐trait models is

(4) $$\left.y_{i}\right|\mu ,\alpha ,\;\Sigma ∼N\left(\mu +\sum_{j\;=\;1}^{p}Z_{ij}\alpha_{j},\Sigma \right)\;\;\;\;\;\;\;i\;=\;1,\;\;\ldots ,\;\;n$$

where $y_{i}$ is an *m*‐element vector of phenotypes for the *i*th individual, μ is an *m*‐element vector for the overall population mean of *m* traits, *Z_ij_* is the SNP genotype code for the *i*th individual at the *j*th marker, **α** *_j_* is an *m*‐element vector for the effects of the *j*th SNP marker on all *m* traits, and **Σ** is an *m* × *m* covariance matrix of the residual effects.

MT‐BayesA uses the genetic correlation between two or more traits to potentially improve the accuracy of prediction (Jia & Jannink, 2012). The MT‐BayesA matrix is an antedependence‐based model that uses correlations between traits as well as SNP effects simultaneously to improve prediction accuracy (Jiang et al., 2015). The standard GS models assume that marker effects are independently distributed; however, this is not always the case, especially when adjacent SNPs are in high LD with the same QTL (Jiang et al., 2015). The antedependence model considers the potential nonstationary correlations between SNP effects near a QTL (Yang & Tempelman, 2012). MT‐BayesA and MT‐BayesA matrix models were fitted using C language programs developed by Jiang et al. (2015).

### 2.5 Genomic prediction scenarios

Three prediction scenarios were evaluated in this study: within population, across population, and across environment. Within‐population genomic predictions were made in each population using a five‐fold cross‐validation design. In a five‐fold cross‐validation, each population was randomly divided into five groups of approximately equal size. In each fold, four groups were combined and used as the TP, and predictions were made for the remaining group. This was repeated five times until predictions were made for each of the five groups. Across‐population genomic predictions were made by using the LDP as the TP to make predictions for the LR‐01 and LR‐11 populations. LR‐01 was used as the TP to make prediction for LR‐11 and vice versa. Across‐environment predictions were made with the reaction norm model using the CV1 and CV2 approaches described above. Across‐population predictions were made using 9394, 24,395, and 39,297 markers that were common between the LDP and LR‐11, the LDP and LR‐01, and LR‐01 and LR‐11, respectively. Within‐population and across‐environment genomic predictions were made using the 24,395 markers in the LDP and 39,297 markers in LR‐01 and LR‐11. The accuracy of all models was determined as Pearson's correlation (*r*) between the predicted values and observed phenotypes of lines in the validation set. For the reaction norm models, prediction accuracy was computed within each environment and fold. The same cross‐validation folds were used for ST‐GS, GS+GWAS, and MT‐GS models for a reliable comparison of their accuracy. Inferences for all Bayesian models were based on 30,000 iterations obtained after discarding 10,000 samples as a burn‐in.

## 3 RESULTS

The distribution of all traits followed an approximately normal distribution in the LDP and LR‐11, but it was slightly skewed toward higher values in LR‐01 because some lines in LR‐01 were susceptible to residue of a herbicide that had been applied the previous fall, which possibly led to delayed flowering and maturity (Supplemental Figure S1). There were strong positive correlations (*r* ≥ .72, *P* = .001) among DTF, VEG, DTS, and DTM in the LDP and LR‐11 populations (Supplemental Figures 1A and 1B). Reproductive period was moderately correlated with DTM but showed weak correlations with the other traits in the LDP and LR‐11. In LR‐01, VEG was strongly correlated with DTF (*r* = .83, *P* = .001) but weakly correlated with the other traits (Supplemental Figure 1c). There was also a strong correlation among DTS, DTM, and REP in LR‐01 (*r* ≥ .76, *P* = .001). A broad range of heritability estimates was obtained for all traits in each population (Table 1). The highest heritability was obtained for VEG in the LDP and LR‐11 and for REP in LR‐01. Heritability of REP was the lowest in the LDP and LR‐11.

**TABLE 1:** Broad‐sense heritability estimates for five traits based on data from the respective number of environments in the lentil diversity panel (LDP) and recombinant inbred lines LR‐01 and LR‐11 populations

	LDP		LR‐01		LR‐11	
Trait^a^	Environments	Heritability	Environments	Heritability	Environments	Heritability
DTF	5	.81	2	.56	4	.63
VEG	3	.82	2	.54	4	.68
DTS	5	.74	2	.80	2	.34
DTM	5	.62	2	.83	4	.42
REP	5	.29	2	.85	4	.17

a DTF, days to flowering; VEG, vegetative period; DTS, days to swollen pods; DTM, days to maturity; REP, reproductive period.

### 3.1 Within‐population prediction accuracy

#### 3.1.1 Single‐trait prediction accuracy

**FIGURE 1:**
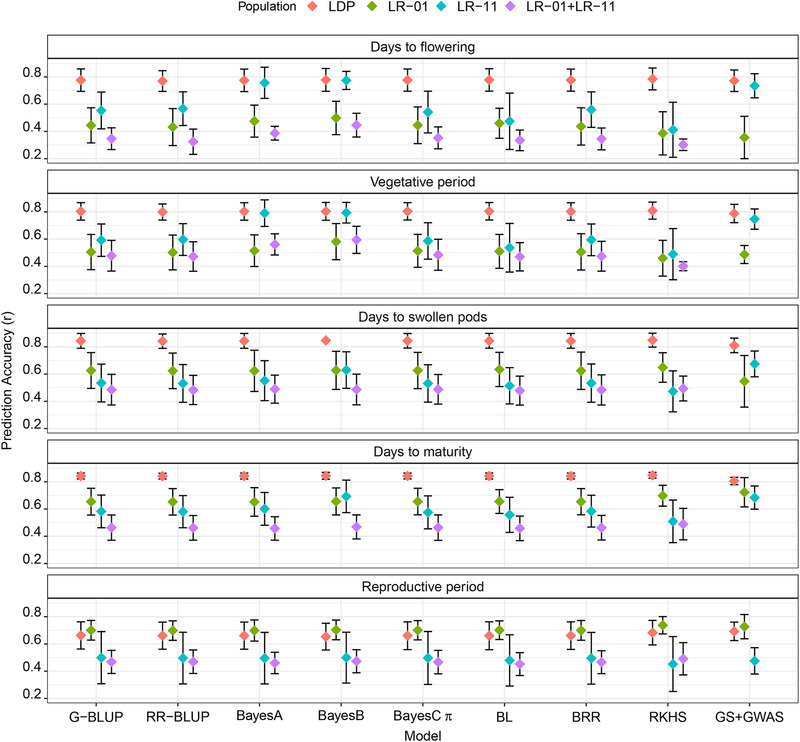
Average within‐population prediction accuracy (based on five‐fold cross‐validation) of nine genomic selection models for five traits measured in the lentil diversity panel (LDP), recombinant inbred lines LR‐01 and LR‐11, and a joint recombinant inbred line population (LR‐01+LR‐11). Vertical bars show standard deviation of the mean. G‐BLUP, genomic best linear unbiased prediction; RR‐BLUP, ridge regression best linear unbiased prediction; BL, Bayesian Lasso; BRR, Bayesian ridge regression; RKHS, Bayesian reproducing kernel Hilbert spaces regression; GS+GWAS, genomic selection model that combines genome‐wide association studies

Single‐trait prediction accuracy varied across traits, regardless of the model. Within‐population prediction accuracies for all traits and models ranged from .65 to .85, .36 to .74, .41 to .79, and .30 to .59 in the LDP, LR‐01, LR‐11, and combined LR‐01 + LR‐11 populations, respectively (Figure 1). Prediction accuracy was lower for REP than for the other traits in the LDP. For each trait, all models showed similar accuracy except for REP, where the RKHS showed 3 to 5% higher accuracy than the other models in the LDP. In LR‐11, the accuracy of BayesA, BayesB, and GS+GWAS was higher for DTF and VEG (Figure 1). GS+GWAS fitted three significant markers from fold‐specific single‐marker regression in the TP as fixed effects while all the remaining markers were fitted as random effects (Supplemental Table S2). GS+GWAS showed higher accuracy than the standard RR‐BLUP, but its accuracy was either similar to or lower than that of BayesB (Figure 1). Similarly, BayesB showed slightly higher accuracy for DTF and VEG in LR‐01 and the joint LR‐01 + LR‐11 populations. Prediction accuracy was high in the LDP compared with the biparental populations for all traits except for REP, for which the highest accuracy was obtained in LR‐01. Combining the LR‐01 and LR‐11 populations resulted in either similar or lower accuracy than the accuracy within each population.

#### 3.1.2 Multiple‐trait prediction accuracy

We compared the accuracies of MT‐BayesA and MT‐BayesA matrix models with ST‐BayesA and the best ST‐GS model with the highest accuracy. In each population, two MT predictions were made using different trait combinations based on their phenotypic correlation (Table 2). In the LDP and LR‐11, the first prediction was made for DTF, VEG, DTS, and DTM (*r* ≥ .72) while the second prediction was made for DTM and REP, with correlations of *r *= .40 and .66 in the LDP and LR‐11, respectively (Supplemental Figures 1A and 1B). In LR‐01, the first prediction was made for DTF and VEG (*r* = .83), while the second prediction was made for DTS, DTM, and REP (*r* ≥ .76) (Supplemental Figure 1C). The accuracy of the MT‐GS models varied depending on the population and traits selected for joint prediction (Table 2). In the LDP, MT‐BayesA and MT‐BayesA matrix models showed 4 to 7% higher accuracy for REP than the accuracy of ST‐BayesA, but accuracies remained the same for all the other traits. MT‐BayesA performed similarly to the best ST‐GS model (RKHS), but MT‐BayesA matrix showed 3% higher accuracy than the accuracy of RKHS for REP (Figure 1, Table 2). In LR‐01, the accuracy of the MT‐GS models was either similar to or lower than the ST‐BayesA except for VEG when it was predicted together with DTF. The accuracy of MT‐BayesA was 10% higher than the accuracy of ST‐BayesA for VEG but similar to the best ST‐GS model (BayesB) in LR‐01. When joint prediction was made for DTM and REP in LR‐11, MT‐BayesA matrix resulted in 15 and 14% higher accuracy for REP than ST‐BayesA and the best ST‐GS model (BayesB), respectively (Table 2). Similarly, the accuracy of the MT‐GS models was higher than the accuracy of ST‐BayesA for DTM, but they had lower accuracy than BayesB in LR‐11.

**TABLE 2:** Average prediction accuracy (from five‐fold cross‐validation) of multiple‐trait (MT)‐BayesA, MT‐BayesA matrix, single‐trait (ST)‐BayesA, and the best ST model in the lentil diversity panel (LDP) and recombinant inbred lines LR‐01 and LR‐11 populations

Population	Prediction^a^	Trait^b^	MT‐BayesA	MT‐BayesA matrix	ST‐BayesA	Best ST model
LDP	1 (*r* ≥ .72)	DTF	.775	.776	.775	.785
		VEG	.800	.799	.803	.809
		DTS	.837	.838	.844	.849
		DTM	.835	.837	.842	.847
	2 (*r *= .40)	DTM	.836	.834	.842	.847
		REP	.686	.706	.661	.683
LR‐01	1 (*r* = .83)	DTF	.498	.428	.475	.499
		VEG	.567	.489	.515	.582
	2 (*r* ≥ .76)	DTS	.544	.501	.623	.648
		DTM	.641	.586	.680	.697
		REP	.682	.647	.699	.737
LR‐11	1 (*r* ≥ .72)	DTF	.695	.726	.757	.774
		VEG	.705	.735	.790	.794
		DTS	.596	.629	.552	.629
		DTM	.620	.643	.601	.693
	2 (*r* = .66)	DTM	.651	.642	.601	.693
		REP	.499	.572	.496	.500

a Joint predictions were made for correlated traits.

b DTF, days to flowering; VEG, vegetative period; DTS, days to swollen pods; DTM, days to maturity; REP, reproductive period.

### 3.2 Across‐population prediction accuracy

**FIGURE 2:**
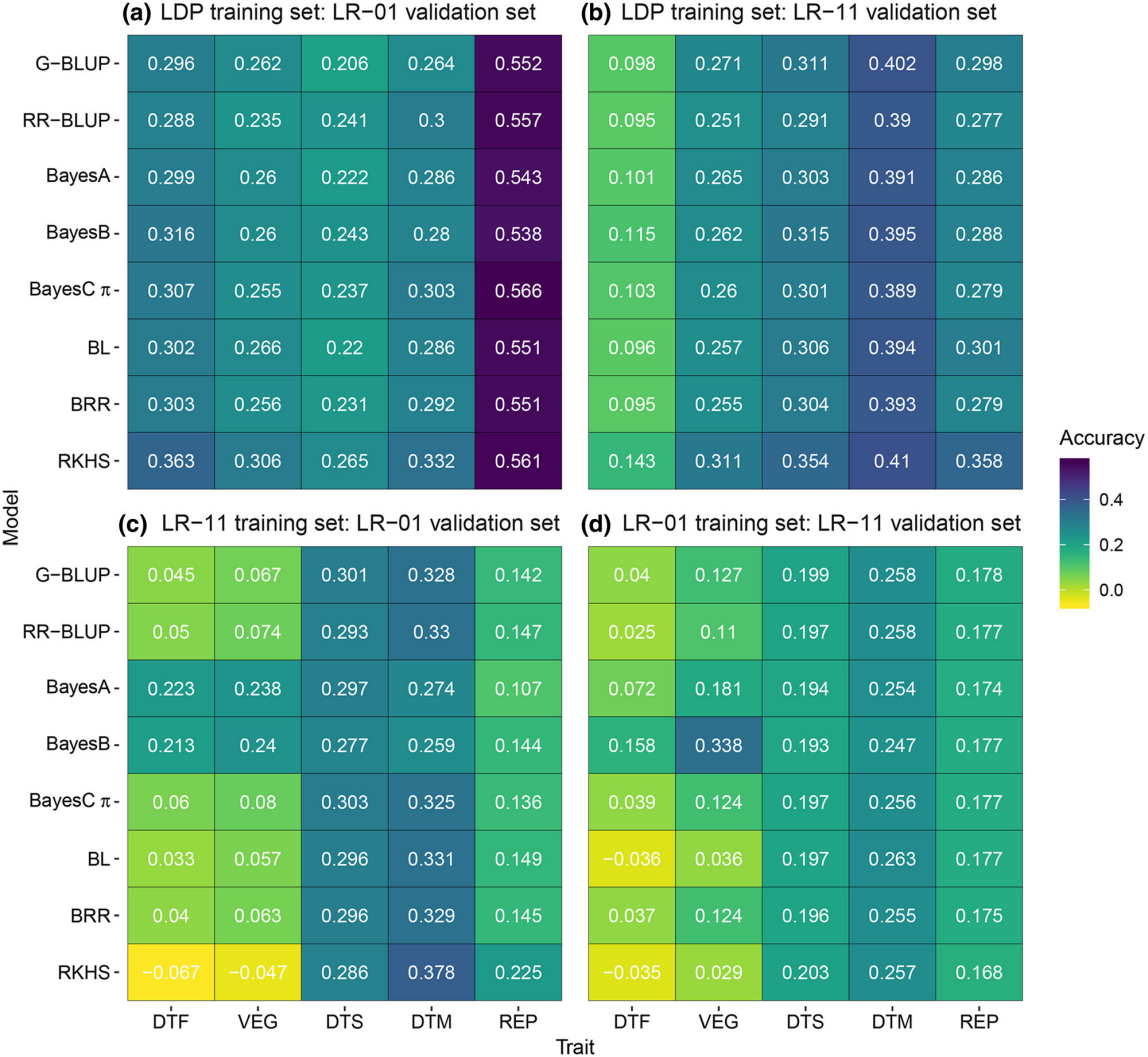
Across‐population prediction accuracy of eight models for days to flowering (DTF), vegetative period (VEG), days to swollen pods (DTS), days to maturity (DTM), and reproductive period (REP): (a) the lentil diversity panel (LDP) was used as a training set and recombinant inbred line LR‐01 was a validation set; (b) the LDP was used as a training set and recombinant inbred line LR‐11 was a validation set; (c) LR‐11 was used as training set and LR‐01 was a validation set; and (d) LR‐01 was used as training set and LR‐11 was a validation set. G‐BLUP, genomic best linear unbiased prediction; RR‐BLUP, ridge regression best linear unbiased prediction; BL, Bayesian Lasso; BRR, Bayesian ridge regression; RKHS, Bayesian reproducing kernel Hilbert spaces regression

Across‐population prediction accuracies were very low for all traits relative to within‐population prediction accuracy except for REP in LR‐01 (Figures 1 and 2). When the LDP was used as the TP to make prediction in LR‐01 and LR‐11, accuracies for all traits ranged from .21 to .57 and from .09 to .41, respectively (Figures 2a and 2b). RKHS consistently resulted in higher across‐population prediction accuracies for all traits when the LDP was used to make prediction in LR‐01 and LR‐11 (Figures 2a and 2b). In LR‐01, prediction accuracy was the highest for REP (ranged from .54 to .56) which also had the highest heritability (Figure 2a). In LR‐11, accuracies were close to zero for DTF but relatively higher accuracies (ranging from .25 to .41) were obtained for the other traits (Figure 2b). When one biparental population was used to make prediction for the other biparental population, accuracies were close to zero or negative for DTF and VEG in all models except in BayesA and BayesB (Figures 2c and 2d). In contrast, slightly higher accuracies (ranging from .26 to .38) were obtained for DTS and DTM in LR‐01 using LR‐11 as the training set (Figure 2c).

### 3.3 Across‐environment prediction accuracy

Across‐environment predictions were made using two cross‐validation designs that simulate prediction for newly developed lines (CV1) and incomplete field trials (CV2). We compared the prediction accuracy of the EG‐G × E model that incorporates GEI with the EG model that included only the main effects of environments and markers without the interaction term. Prediction accuracies varied depending on the cross‐validation design and population. For all traits in the LDP, prediction accuracies of EG‐G × E ranged from .52 to .82 in CV1 and from .63 to .95 in CV2 (Table 3). The lowest accuracies were obtained for REP in both cross‐validation designs. For REP, modeling GEI resulted in 7–30 and 4–35% higher accuracy than EG in CV1 and CV2, respectively (Table 3). Similarly, there was a 4–6% increase in prediction accuracy for DTM when modeling GEI in Sutherland 2016. For all other traits, however, modeling the interaction term did not improve the accuracy of prediction. In LR‐01, accuracies for all traits ranged from .41 to .66 and .63 to .88 in CV1 and CV2, respectively (Table 3). Both models showed similar accuracy for all traits in LR‐01, indicating that there was no benefit of modeling GEI when trait heritability is high. Similarly, prediction accuracies of EG‐G × E in LR‐11 ranged from .19 to .60 and .36 to .89 for all traits in CV1 and CV2, respectively. The lowest accuracies were obtained for REP, which also had low heritability (*h* ^2^ = .17). Similar accuracies were obtained between the two models for all traits in LR‐11 except for REP in CV2, where incorporating the interaction term resulted in 18–66% higher accuracy. This shows that modeling GEI improved the accuracy of prediction for a low‐heritability trait but modeling only the main effects was enough for traits of high heritability. Overall, CV2 consistently resulted in higher accuracy than CV1 for all traits and populations (Table 3).

**TABLE 3:** Across‐environment prediction accuracy (from five‐fold design) for four traits predicted using the EG and EG‐G × E models for cross‐validation CV1 and CV2

	Model EG				Model EG‐G × E			
Environment	DTF	DTM	DTS	REP	DTF	DTM	DTS	REP
	Lentil diversity panel							
CV1^a^								
Rosthern, 2016	.749 (.039)	.766 (.063)	.797 (.036)	.488 (.085)	.734 (.053)	.761 (.038)	.802 (.042)	.524 (.078)
Rosthern, 2017	.802 (.073)	.738 (.061)	.794 (.070)	.558 (.100)	.803 (.074)	.752 (.053)	.798 (.076)	.597 (.104)
Sutherland, 2016	.798 (.074)	.746 (.096)	.807 (.063)	.529 (.099)	.796 (.078)	.777 (.071)	.811 (.059)	.609 (.032)
Sutherland, 2017	.812 (.066)	.769 (.074)	.822 (.078)	.490 (.186)	.819 (.062)	.779 (.034)	.822 (.078)	.638 (.121)
Sutherland, 2018	.787 (.069)	.729 (.088)	.806 (.055)	.585 (.056)	.785 (.077)	.746 (.060)	.821 (.057)	.699 (.058)
CV2								
Rosthern, 2016	.896 (.027)	.856 (.032)	.875 (.026)	.595 (.086)	.901 (.029)	.861 (.037)	.877 (.042)	.632 (.112)
Rosthern, 2017	.943 (.013)	.826 (.033)	.890 (.022)	.625 (.093)	.947 (.011)	.840 (.036)	.915 (.017)	.649 (.083)
Sutherland, 2016	.950 (.018)	.811 (.033)	.922 (.011)	.579 (.100)	.953 (.013)	.857 (.025)	.921 (.019)	.698 (.074)
Sutherland, 2017	.958 (.006)	.864 (.025)	.933 (.018)	.558 (.080)	.950 (.006)	.878 (.023)	.936 (.006)	.720 (.047)
Sutherland, 2018	.923 (.018)	.807 (.055)	.903 (.018)	.526 (.100)	.931 (.032)	.812 (.054)	.925 (.013)	.708 (.047)
	Recombinant inbred line LR‐01							
CV1								
Rosthern, 2018	.484 (.156)	.573 (.086)	.543 (.140)	.608 (.053)	.485 (.149)	.576 (.086)	.548 (.139)	.611 (.054)
Sutherland, 2018	.408 (.147)	.613 (.074)	.552 (.149)	.654 (.066)	.406 (.150)	.615 (.073)	.559 (.141)	.656 (.065)
CV2								
Rosthern, 2018	.637 (.187)	.831 (.021)	.836 (.082)	.849 (.033)	.638 (.187)	.850 (.056)	.837 (.081)	.854 (.062)
Sutherland, 2018	.631 (.075)	.870 (.033)	.765 (.102)	.882 (.033)	.630 (.074)	.862 (.031)	.767 (.102)	.867 (.040)
	Recombinant inbred line LR‐11							
CV1								
Rosthern, 2017	.587 (.120)	.499 (.127)	−b	.295 (.163)	.585 (.123)	.499 (.128)	−	.299 (.162)
Rosthern, 2018	.457 (.138)	.331 (.052)	.428 (.102)	.296 (.162)	.456 (.139)	.331 (.047)	.430 (.103)	.299 (.157)
Sutherland, 2017	.595 (.153)	.575 (.110)	−	.194 (.165)	.595 (.157)	.574 (.106)	−	.192 (.166)
Sutherland, 2018	.512 (.211)	.507 (.161)	.440 (.154)	.393 (.146)	.514 (.210)	.509 (.155)	.442 (.153)	.395 (.145)
CV2								
Rosthern, 2017	.891 (.044)	.738 (.050)	−	.370 (.327)	.890 (.045)	.736 (.049)	−	.521 (.154)
Rosthern, 2018	.736 (.060)	.545 (.163)	.597 (.146)	.383 (.252)	.736 (.060)	.546 (.162)	.597 (.147)	.453 (.135)
Sutherland, 2017	.889 (.042)	.794 (.087)	−	.220 (.304)	.889 (.042)	.792 (.090)	−	.364 (.212)
Sutherland, 2018	.892 (.053)	.770 (.114)	.640 (.126)	.326 (.173)	.892 (.054)	.769 (.14)	.639 (.126)	.442 (.131)

*Notes*: Average and standard deviation (in parentheses). DTF, days to flowering; VEG, vegetative period; DTS, days to swollen pods; DTM, days to maturity; REP, reproductive period.

a CV1, prediction for lines that were not evaluated in any of the environments; CV2, prediction for lines that were evaluated in some of the environments.

b Data not available.

## 4 DISCUSSION

This study evaluated the accuracy of ST‐GS models, MT‐GS models, a model that included significant markers from GWAS as fixed effects, and a model that accounted for GEI using a lentil diversity panel and two RIL populations. Moreover, within‐population, across‐population, and across‐environment predictions were tested to simulate scenarios that breeders may face when implementing GS. Highly variable prediction accuracies were obtained depending on the trait, population, prediction scenario, and statistical model used.

### 4.1 Within‐population genomic prediction

Nine ST‐GS and two MT‐GS models were used to make within‐population genomic predictions based on a five‐fold cross‐validation design. Prediction accuracies ranged from .65 to .85, .36 to .74, .41 to .79, and .30 to .59 for all traits in the LDP, LR‐01, LR‐11, and combined LR‐01 + LR‐11 populations, respectively (Figure 1). The degree of genetic relationship between the training and validation sets is an important factor that affects the accuracy of GS prediction, with the accuracy being higher for lines that are closely related (Habier, Fernando, & Dekkers, 2007; Riedelsheimer et al., 2013). Lines in the biparental populations are closely related compared with the lines in the LDP, but accuracies were generally higher in the LDP than the biparental populations. This could be because the number of lines in the LDP is nearly three times that of the biparental populations. Training population size is another important factor that affects the accuracy of genomic predictions. Increasing the TP size increases the accuracy of prediction because it provides more data to estimate marker effects (Asoro et al., 2011; Saatchi, Miraei‐Ashtiani, Javaremi, Moradi‐Shahrebabak, & Mehrabani‐Yeghaneh, 2010; VanRaden et al., 2009). All models showed similar accuracy in the LDP except for RKHS, which showed 3–5% higher accuracy than the other models for REP. BayesA, BayesB, and GS+GWAS resulted in higher accuracy for DTF and VEG in LR‐11 (Figure 1). In theory, RR‐BLUP is expected to have higher accuracy for traits controlled by many QTL with small effects, while Bayesian models have higher accuracy when few QTL with large effects control most of the phenotypic variance (Lorenz et al., 2011). The higher accuracy of BayesA and BayesB in LR‐11 could be due to the presence of large‐effect QTL controlling DTF and VEG.

The accuracy of RKHS was slightly higher for REP in the LDP but it resulted in similar or lower accuracy than the other models for all traits (Figure 1). This suggests that the contribution of nonadditive genetic effects to the total genetic variance is negligible in the populations used for this study. Previous studies in wheat and maize also reported no benefit of models accounting for nonadditive effects over simple additive models (Lorenzana & Bernardo, 2009; Sallam, Endelman, Jannink, & Smith, 2015; Zhao, Zeng, Fernando, & Reif, 2013). In contrast, improved prediction accuracies were reported using RKHS and models that incorporate epistasis (Crossa et al., 2010; Pérez‐Rodríguez et al., 2012). This suggests that the benefit of accounting for nonadditive effects in GS may vary depending on the trait and population type.

GS+GWAS fitted up to three markers that are significantly associated with traits as fixed effects (Supplemental Table S2). There was no benefit of fitting significant markers as fixed effects in the LDP because all markers explained less than 5% of the phenotypic variance (Supplemental Table S2). GS+GWAS works best for traits with one or more medium to large effect QTL segregating in the population but it has no advantage if a trait has no significant GWAS peaks (Spindel et al., 2016). Bernardo (2014), suggested that major QTL should be fitted as having fixed effects in GS, especially if a few major QTL are present and if each QTL explains more than 10% of the genetic variance. Including up to three significant markers as a fixed effect in GS+GWAS resulted in 18 to 30% higher accuracy than the standard RR‐BLUP for DTF, VEG, DTS ,and DTM in LR‐11 (Figure 1). Similarly, 4 and 11% higher accuracy was obtained for REP and DTM, respectively, in LR‐01. However, no improvement in accuracy was observed for the other traits in LR‐01 and LR‐11. Spindel et al. (2016) showed that GS+GWAS performed better than the standard RR‐BLUP in all cases, and up to 30% higher accuracy was obtained for flowering time of rice (*Oryza sativa* L.), which had a large GWAS peak. Although GS+GWAS gave higher accuracies than the standard RR‐BLUP in LR‐01 and LR‐11, its accuracy was either similar to or lower than BayesB, indicating that variable selection models such as BayesB can be equally effective to account for large‐effect QTL in GS.

Combining LR‐01 and LR‐11 to increase the TP size did not improve the accuracy of prediction for all traits (Figure 1). Previous studies in wheat and barley (*Hordeum vulgare* L.) also indicated that prediction accuracies did not improve when unrelated populations from different breeding programs were merged to increase TP size (Charmet et al., 2014; Lorenz, Smith, & Jannink, 2012). Unrelated populations may have different LD phases, and combining them into a single TP reduces the overall LD (Goddard, 2012). Moreover, combining multiple populations may create strong population structure and allele frequency differences between subpopulations, which reduces GS accuracy (Riedelsheimer et al., 2013). The standard GS models assume that marker effects are constant across subpopulations and fail to account for differences in allele frequency due to population structure. Therefore, models that account for population structure need to be tested when unrelated populations are combined to increase TP size.

Similar accuracies were obtained between MT‐GS and ST‐GS models when joint predictions were made for correlated high heritability traits (Tables 1 and 2). However, the accuracy of MT‐BayesA matrix increased by 3 and 14% for REP when it was predicted with DTM in the LDP and LR‐11, respectively (Table 2). Jiang et al. (2015) also showed that the prediction accuracy of the simplified version of the MT‐BayesA matrix model was 2.8–8.6% higher than that of the best ST‐GS model. Previous studies based on simulated data showed that MT‐GS models have higher accuracy for a low heritability trait (*h* ^2^ = .1) jointly predicted with a correlated high heritability trait (*h* ^2^ ≥ .5) (Hayashi & Iwata, 2013; Jia & Jannink, 2012; Jiang et al., 2015). However, no improvement in accuracy was observed compared with ST‐GS models for traits of high heritability and in the absence of genetic correlation between traits (Hayashi & Iwata, 2013; Jia & Jannink, 2012; Jiang et al., 2015).

### 4.2 Across‐population genomic prediction

Variable across‐population prediction accuracies were obtained depending on the trait and population used as TP. Overall, across‐population prediction accuracies were low relative to within‐population prediction accuracy for all traits except for REP in LR‐01 (Figures 1 and 2). The prediction accuracy was the highest (range .54–.56) for REP, which indicates that moderate across‐population prediction can be made for highly heritable traits (Figure 2a). Prediction in LR‐01 and LR‐11 ranged from .21 to .57 and .09 to .41, respectively, when the LDP was used as the TP (Figures 2a and 2b). When one biparental population was used to predict the other biparental population, accuracies were close to zero or negative for DTF and VEG in all models except in BayesA and BayesB (Figures 2c and 2d). Previous studies in wheat and maize also reported mean accuracies of zero or negative values when an unrelated TP was used to make prediction for an independent population (Charmet et al., 2014; Crossa et al., 2014; Riedelsheimer et al., 2013; Windhausen et al., 2012). Genomic selection models use both the genetic relationships among individuals and LD between markers and QTL to improve prediction accuracy (Habier et al., 2007). Across‐population prediction not only requires strong LD but similar linkage phases in each population (Goddard & Hayes, 2007). When the TP is unrelated to the breeding population, marker effects estimated in one population cannot be transferred to the other population due to differences in allele frequency and LD phase resulting in low prediction accuracy (Bassi et al., 2016; Windhausen et al., 2012). In plant breeding, early generation nurseries are comprised of different families from many crosses, and the most attractive application of GS is to estimate GEBVs of lines in these families based on marker effects estimated from an independent population. However, the results of this study, as well as previous studies, showed that GS has very low accuracy for such applications. Based on a simulation study, Meuwissen (2009) suggested that a substantially higher marker density and TP size is required for accurate prediction of GEBVs in unrelated individuals. Therefore, across‐population prediction should not be considered unless the two populations are closely related or the size of the TP is very large.

### 4.3 Across‐environment genomic prediction

Across‐environment predictions were made using reaction norm models that incorporated the main and interaction terms of environments and markers (Jarquín et al., 2014). Genotype × environment interaction is an important factor that affects both phenotypic and genomic selection accuracy. Prediction accuracies in CV1 ranged from .49 to .82, .41 to .66, and .19 to .60 in the LDP, LR‐01, and LR‐11, respectively (Table 3). Prediction accuracies in CV2 were consistently higher than those in CV1 and ranged from .53 to .96, .63 to .88, and .22 to .89 in the LDP, LR‐01, and LR‐11, respectively. Similar results were obtained in previous studies that used the same cross‐validation designs in wheat and cotton (*Gossypium hirsutum* L.) (Burgueño et al., 2012; Crossa et al., 2015; Jarquín et al., 2014; Jarquín et al., 2017; Lopez‐Cruz et al., 2015; Pérez‐Rodríguez et al., 2015). This is because CV2 allows borrowing of information for the same line across environments. Modeling the interaction term improved the accuracy of prediction for REP by 4–35 and 18–66% in the LDP and LR‐11, respectively (Table 3). But for all other traits, modeling GEI resulted in similar accuracies to the model that included only the main effects of markers and environments. Previous studies in wheat and maize showed that modeling GEI improves the accuracy of genomic predictions (Crossa et al., 2015; Cuevas et al., 2017; Jarquín et al., 2014; Jarquín et al., 2017; Lopez‐Cruz et al., 2015). Jarquín et al. (2014) used the reaction norm model to predict the grain yield of wheat and reported a 35% increase in accuracy when adding interaction terms between markers and environments. Recently, Jarquín et al. (2017) showed that modeling GEI resulted in 16 to 82% higher accuracy than a baseline model that did not include the GEI term. Other studies in wheat and cotton that used the reaction norm model with pedigrees instead of molecular markers also obtained the highest prediction accuracy when a GEI term was included in the model (Pérez‐Rodríguez et al., 2015; Sukumaran, Crossa, Jarquín, & Reynolds, 2017).

## 5 CONCLUSIONS

This research provides the first empirical evidence of GS in lentil. Comparison of different models and approaches showed that most ST‐GS models have similar accuracy in the absence of large effect QTL underlying the traits, but BayesB is the best model when QTL with relatively large effects are present. Genomic selection models that accounted for GEI and MT‐GS models improved the accuracy of prediction for a low heritability trait, but they have no advantage when trait heritability is high. Within‐population and across‐environment genomic predictions resulted in moderate to high accuracies, but across‐population prediction accuracies were very low. This suggests that GS can be implemented in lentil to make within‐population or across‐environment predictions, but across‐population prediction should not be considered when the population size is small.

## AUTHOR CONTRIBUTIONS

TAH generated phenotypic data, performed all statistical analyses, and wrote the manuscript. TH, DW, SN generated phenotypic data, and edited the manuscript. LR generated the SNP data set. AV designed the experiment and provided germplasm materials. KEB designed the experiment, supervised the project, and edited the manuscript. All authors read and approved the submitted version.

## CONFLICT OF INTEREST

The authors declare that they have no conflict of interest.

## Supporting information


**Supplemental Fig. S1**. Frequency distribution and pairwise correlations among days to flowering (DTF), vegetative period (VEG), days to swollen pods (DTS), days to maturity (DTM), and reproductive period (REP) in (A) LDP, (B) LR‐11, and (C) LR‐01 populations.

**Supplemental Table S1. Description of trials conducted in each environment and coefficient of variation for traits measured in each population and environment**.

**Supplemental Table S2. List of significant markers (from fold specific GWAS in the LDP and single marker regression in LR‐01 and LR‐11) fitted as a fixed effect in the GS+GWAS model**.
