# Supplementary material for: Genomic selection for lentil breeding: Empirical evidence
Source: Plant Genome. 2020 Mar 27;13(1):e20002. doi: 10.1002/tpg2.20002 (PMC12807041; doi:10.1002/tpg2.20002)
Supplement: Supplementary file 1 [file TPG2-13-e20002-s001.pdf]

# Genomic Selection for Lentil Breeding: Empirical Evidence

Teketel A. Haile[1], Taryn Heidecker[1], Derek Wright[1], Sandesh Neupane[1], Larissa Ramsay[1], Albert Vandenberg[1], and Kirstin E. Bett[1]*

[1]Department of Plant Sciences, University of Saskatchewan, 51 Campus Drive, Saskatoon, SK, S7N 5A8, Canada.

*Corresponding author

E-mail: k.bett@usask.ca

(A)

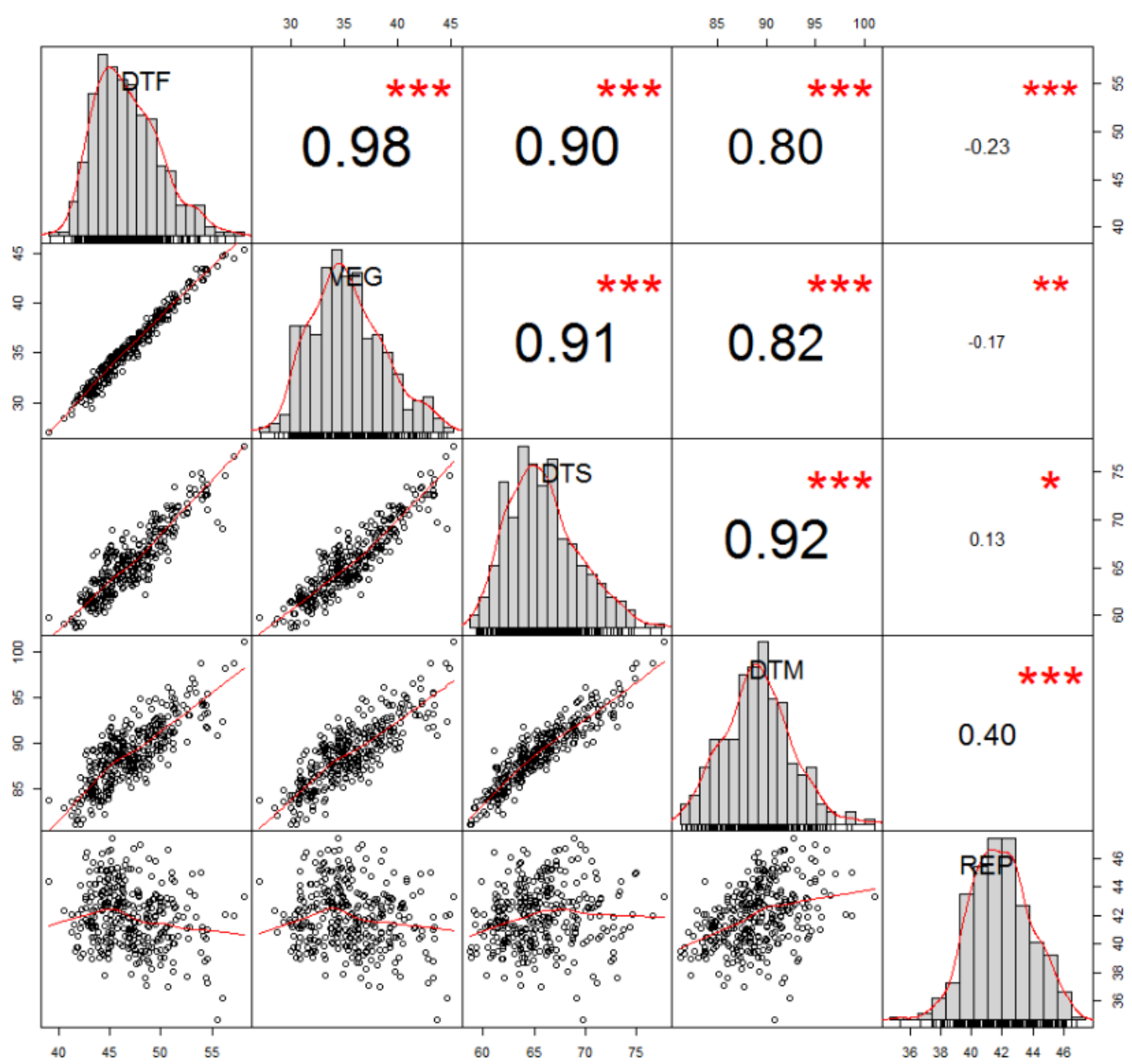

(B)

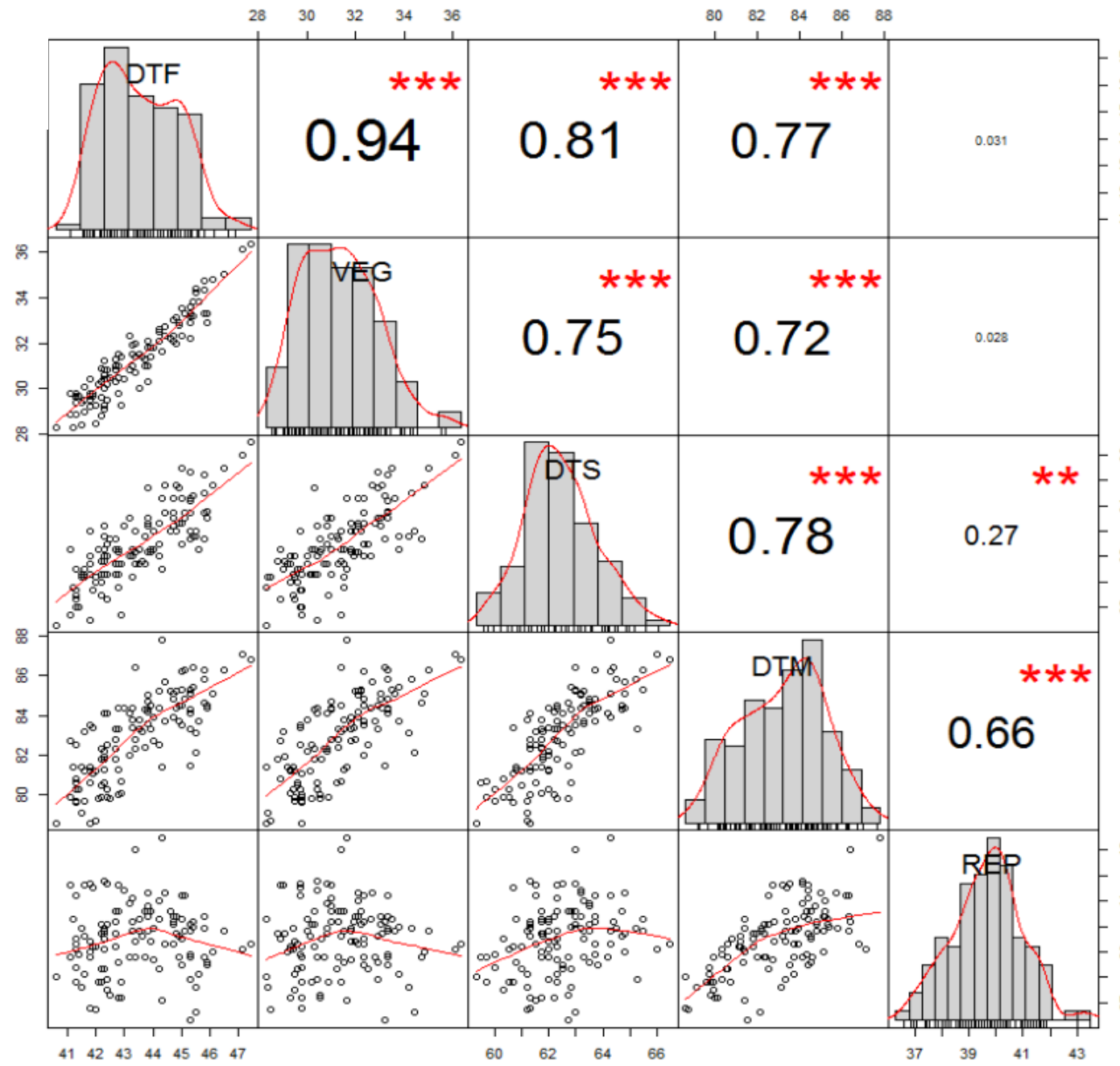


(C)

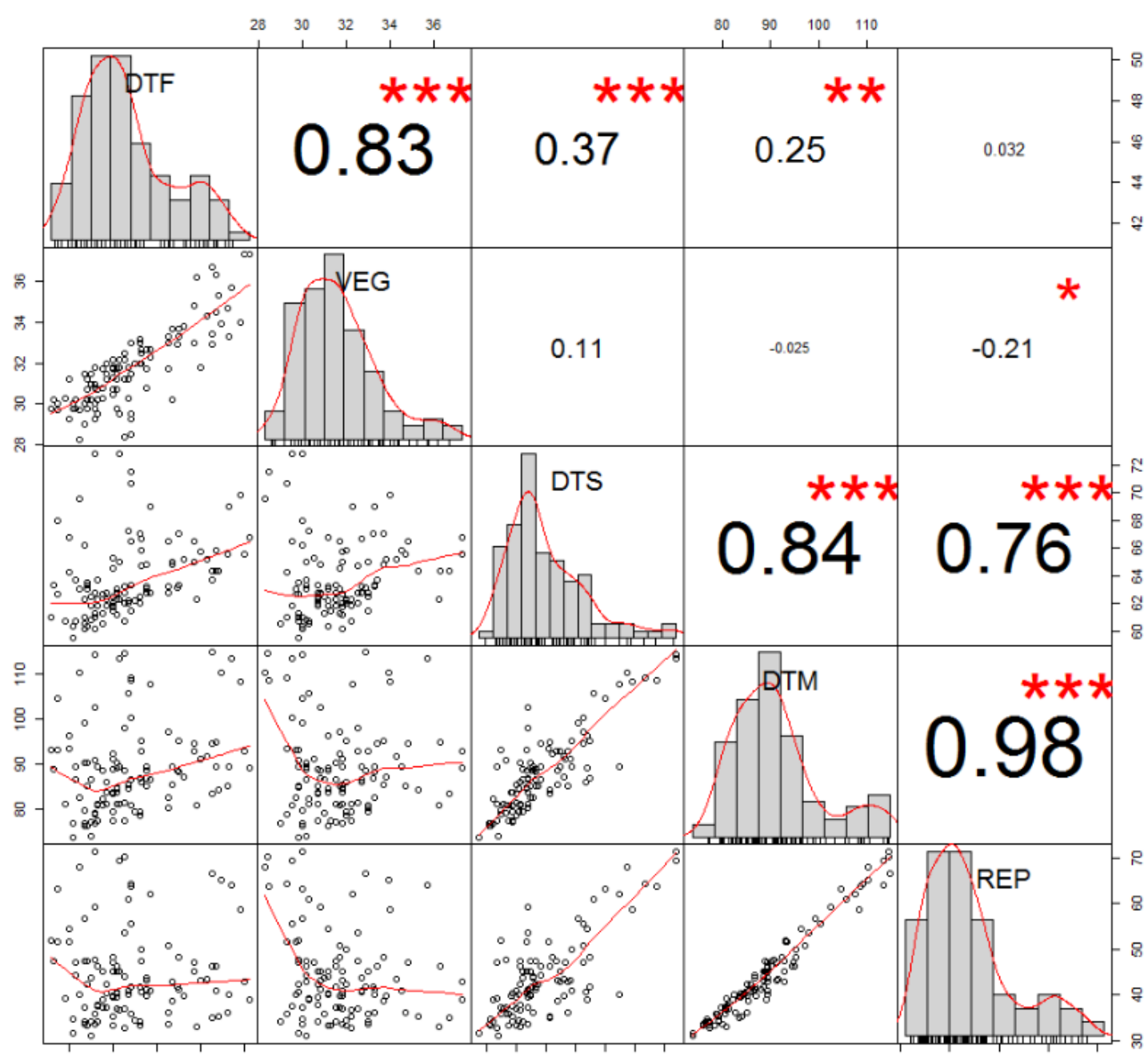


**Supplemental Fig. 1.** Frequency distribution and pairwise correlations among days to flowering (DTF), vegetative period (VEG), days to swollen pods (DTS), days to maturity (DTM), and reproductive period (REP) in A) LDP, B) LR-11, and C) LR-01 populations. *, **, and *** indicate significance at the 0.05, 0.01, and 0.001 probability levels, respectively.
