# Supplementary material for: Genomic selection for lentil breeding: Empirical evidence
Source: Plant Genome. 2020 Mar 27;13(1):e20002. doi: 10.1002/tpg2.20002 (PMC12807041; doi:10.1002/tpg2.20002)
Supplement: Supplementary file 2 [file TPG2-13-e20002-s002.pdf]

Genomic Selection for Lentil Breeding: Empirical Evidence

Teketel A. Haile[1], Taryn Heidecker[1], Derek Wright[1], Sandesh Neupane[1], Larissa Ramsay[1], Albert Vandenberg[1], and Kirstin E. Bett[1]*

[1]Department of Plant Sciences, University of Saskatchewan, 51 Campus Drive, Saskatoon, SK, S7N 5A8, Canada.

*Corresponding author

E-mail: k.bett@usask.ca

Supplemental Table 1. Description of trials conducted in each environment and coefficient of variation for traits measured in each population and environment.

Population	Environment	Number of replications	DTF†	VEG	DTS	DTM	REP
			Coefficient of variation (%)				
LDP (324 lines)	Rosthern 2016	3	6.5	−	6.2	3.4	6.1
	Rosthern 2017	3	7.9	10.3	6.2	4.0	7.6
	Sutherland 2016	3	7.6	−	7.1	4.3	7.3
	Sutherland 2017	3	9.8	13.5	5.7	3.7	8.7
	Sutherland 2018	3	6.7	9.7	6.5	6.4	10.3
LR-01 (110 lines)	Rosthern 2018	3	6.2	7.0	6.2	10.7	21.2
	Sutherland 2018	3	5.7	7.6	5.7	12.1	23.7
LR-11 (120 lines)	Rosthern 2017	3	4.5	6.0	−	3.6	6.9
	Rosthern 2018	3	3.9	6.5	3.4	3.7	6.4
	Sutherland 2017	3	5.7	8.1	−	2.9	5.2
	Sutherland 2018	3	3.3	6.3	3.4	4.7	8.1

† DTF, days to flowering; VEG, vegetative period; DTS, days to swollen pods; DTM, days to maturity; REP, reproductive period.
−, data not available.
